# Genome-Wide Profiling of Yeast DNA:RNA Hybrid Prone Sites with DRIP-Chip

**DOI:** 10.1371/journal.pgen.1004288

**Published:** 2014-04-17

**Authors:** Yujia A. Chan, Maria J. Aristizabal, Phoebe Y. T. Lu, Zongli Luo, Akil Hamza, Michael S. Kobor, Peter C. Stirling, Philip Hieter

**Affiliations:** 1 Michael Smith Laboratories, University of British Columbia, Vancouver, Canada; 2 Centre for Molecular Medicine and Therapeutics, Child and Family Research Institute, Vancouver, Canada; 3 Wine Research Centre, University of British Columbia, Vancouver, Canada; 4 Department of Medical Genetics, University of British Columbia, Vancouver, Canada; 5 Terry Fox Laboratory, British Columbia Cancer Agency, Vancouver, Canada; Stanford University School of Medicine, United States of America

## Abstract

DNA:RNA hybrid formation is emerging as a significant cause of genome instability in biological systems ranging from bacteria to mammals. Here we describe the genome-wide distribution of DNA:RNA hybrid prone loci in *Saccharomyces cerevisiae* by DNA:RNA immunoprecipitation (DRIP) followed by hybridization on tiling microarray. These profiles show that DNA:RNA hybrids preferentially accumulated at rDNA, Ty1 and Ty2 transposons, telomeric repeat regions and a subset of open reading frames (ORFs). The latter are generally highly transcribed and have high GC content. Interestingly, significant DNA:RNA hybrid enrichment was also detected at genes associated with antisense transcripts. The expression of antisense-associated genes was also significantly altered upon overexpression of RNase H, which degrades the RNA in hybrids. Finally, we uncover mutant-specific differences in the DRIP profiles of a Sen1 helicase mutant, RNase H deletion mutant and Hpr1 THO complex mutant compared to wild type, suggesting different roles for these proteins in DNA:RNA hybrid biology. Our profiles of DNA:RNA hybrid prone loci provide a resource for understanding the properties of hybrid-forming regions *in vivo*, extend our knowledge of hybrid-mitigating enzymes, and contribute to models of antisense-mediated gene regulation. A summary of this paper was presented at the 26^th^ International Conference on Yeast Genetics and Molecular Biology, August 2013.

## Author Summary

RNA processing factors are mutated in human cancers, inherited developmental disorders and neurodegenerative syndromes. Defects in RNA processing have been associated with increased levels of mutations and DNA damage in part via the formation of DNA:RNA hybrids. Although it is likely that specific regions of the genome are more prone to DNA:RNA hybrid formation, a map of hybrid-prone regions is not available. In this study, we describe the genome-wide distribution of DNA:RNA hybrids in both normal and mutant *Saccharomyces cerevisiae* cells. The resulting profiles contribute to both our understanding of the general properties of hybrid-forming loci and to our knowledge of hybrid-mitigating enzymes. Interestingly, significant DNA:RNA hybrid enrichment was detected at genes associated with antisense transcription. We show that overexpression of RNase H, which degrades the RNA in hybrids, significantly affects the expression of genes associated with antisense transcripts. These findings support a role for DNA:RNA hybrids in regulation of gene expression by antisense transcripts.

## Introduction

Elevated DNA:RNA hybrid formation due to defects in RNA processing pathways leads to genome instability and replication stress across species [1]–[7]. R loops threaten genome stability and often form under abnormal conditions where nascent mRNA is improperly processed or RNA half-life is increased, resulting in RNA that can hybridize with template DNA, displacing the non-transcribed DNA strand [8]. A recent study also found that hybrid formation can occur in *trans* via Rad51-mediated DNA-RNA strand exchange [9]. Persistent R loops pose a major threat to genome stability through two mechanisms. First, the exposed non-transcribed strand is susceptible to endogenous DNA damage due to the increased exposure of chemically reactive groups. The second, more widespread mechanism, identified in *Escherichia coli*, *Saccharomyces cerevisiae*, *Caenorhabditis elegans* and human cells, involves the R loops and associated stalled transcription complexes, which block DNA replication fork progression [3], [4], [8], [10], [11]. R loop-mediated instability is an area of great interest primarily because genome instability is considered an enabling characteristic of tumor formation [12]. Moreover, mutations in RNA splicing/processing factors are frequently found in human cancer, heritable diseases like Aicardi-Goutieres syndrome, and a degenerative ataxia associated with Senataxin mutations [13]–[17].

To avoid the deleterious effects of R loops, cells express enzymes for the removal of abnormally formed DNA:RNA hybrids. In *S. cerevisiae*, *RNH1* and *RNH201*, each encoding RNase H are responsible for one of the best characterized mechanisms for reducing R loop formation by enzymatically degrading the RNA in DNA:RNA hybrids [8]. Another extensively studied anti-hybrid factor is the THO/TREX complex which functions to suppress hybrid formation at the level of transcription termination and mRNA packaging [4], [11], [18], [19]. In addition, the Senataxin helicase, yeast Sen1, plays an important role in facilitating replication fork progress through transcribed regions and unwinding RNA in hybrids to mitigate R loop formation and RNA polymerase II transcription-associated genome instability [5], [20]. Several additional anti-hybrid mechanisms have also been identified including topoisomerases and other RNA processing factors [2], [6], [7], [9], [21]–[23].

To add to the complexity of DNA:RNA hybrid management in the cell, hybrids also occur naturally and have important biological functions [24]. In human cells, R loop formation facilitates immunoglobulin class switching, protects against DNA methylation at CpG island promoters and plays a key role in pause site-dependent transcription termination [25]–[28]. Transcription of telomeres by RNA polymerase II also produces telomeric repeat-containing RNAs (TERRA), which associate with telomeres and inhibit telomere elongation in a DNA:RNA hybrid-dependent fashion [29]–[31]. Noncoding (nc)RNA such as antisense transcripts, perform a regulatory role in the expression of sense transcripts that may involve R loops [32]. The proposed mechanisms of antisense transcription regulation are not clearly understood and involve different modes of action specific to each locus. Current models include chromatin modification resulting from antisense-associated transcription, antisense transcription modulation of transcription regulators, collision of sense and antisense transcription machineries and antisense transcripts expressed in *trans* interacting with the promoter for sense transcription [32]–[40]. More recently, studies in *Arabidopsis thaliana* found an antisense transcript that forms R loops, which can be differentially stabilized to modulate gene regulation [41]. Similarly, in mouse cells the stabilization of an R loop was shown to inhibit antisense transcription [42].

Here we describe, for the first time, a genome-wide profile of DNA:RNA hybrid prone loci in *S. cerevisiae* by DNA:RNA immunoprecipitation followed by hybridization on tiling microarrays (DRIP-chip). We found that DNA:RNA hybrids occurred at highly transcribed regions in wild type cells, including some identified in previous studies. Remarkably, we observed that DNA:RNA hybrids were significantly associated with genes that have corresponding antisense transcripts, suggesting a role for hybrid formation at these loci in gene regulation. Consistently, we found that genes whose expression was altered by overexpression of RNase H were also significantly associated with antisense transcripts. A small-scale cytological screen found that diverse RNA processing mutants had increased hybrid formation and additional DRIP-chip studies revealed specific hybrid-site biases in the RNase H, Sen1 and THO complex subunit Hpr1 mutants. These genome-wide analyses enhance our understanding of DNA:RNA hybrid-forming regions *in vivo*, highlight the role of cellular RNA processing activities in suppressing hybrid formation, and implicate DNA:RNA hybrids in control of a subset of antisense regulated loci.

## Results

### The genomic distribution of DNA:RNA hybrids

DNA:RNA hybrids have been previously immunoprecipitated at specific genomic sites such as rDNA, selected endogenous loci, and reporter constructs [2], [5]. Subsequently, DRIP coupled with deep sequencing in human cells has demonstrated the prevalence of R loops at CpG island promoters with high GC skew [26]. To investigate the global profile of DNA:RNA hybrid prone loci in a tractable model, we performed genome-wide DRIP-chip analysis of wild type *S. cerevisiae* (ArrayExpress E-MTAB-2388) using the S9.6 monoclonal antibody which specifically binds DNA:RNA hybrids, as characterized previously [43], [44]. DRIP-chip profiles were generated in duplicate (spearman's ρ = 0.78 when comparing each of over 2 million probes after normalization and data smoothing, **Supplementary Figure S1**) and normalized to a no antibody control.

**Figure 1:**
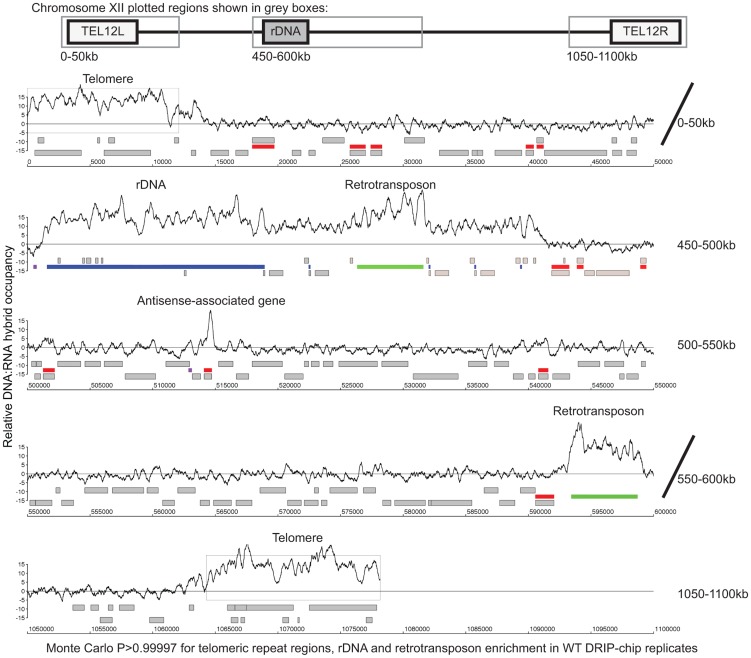
Genome-wide profile of DNA:RNA hybrids in wild type yeast revealed enrichment at rDNA, telomeres, retrotransposons and a subset of genes. DRIP-chip chromosome plot of DNA:RNA hybrids in the rDNA region and telomeric ends of chromosome XII. The black line represents the average of two wild type replicates. Bars indicate ORFs (grey), rDNA (purple), retrotransposons (green) or genes associated with an antisense transcript (red) [51], [54]). Grey boxes delineate telomeric repeat regions. Y-axis indicates relative occupancy of DNA:RNA hybrids. X-axis indicates chromosomal coordinates. P indicates probability of observing a number of enriched features by random chance below what was observed (P>0.99997).

**Figure 2:**
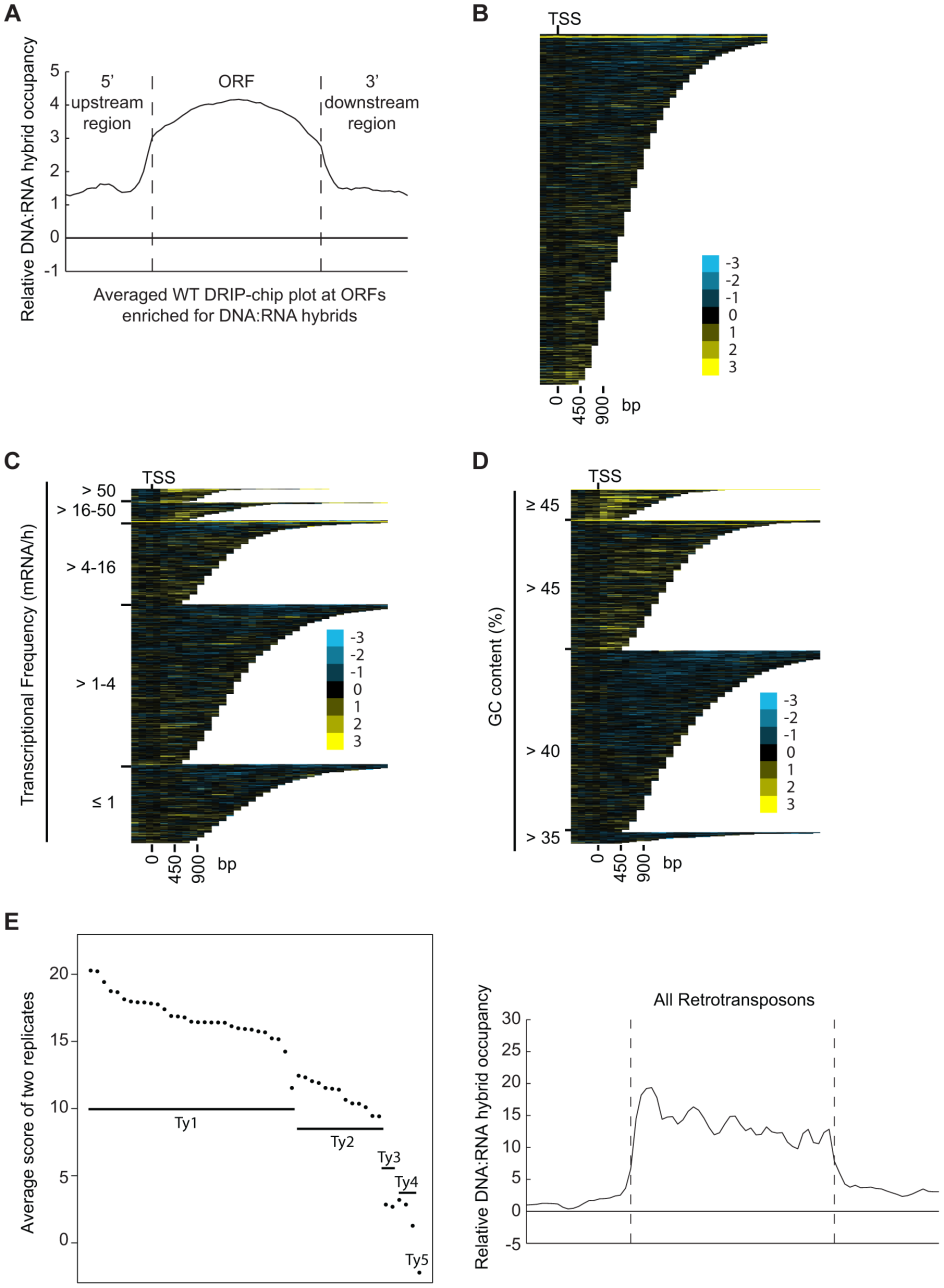
DNA:RNA hybrids are enriched at protein-encoding genes and retrotransposons of higher transcriptional frequency. (A) Average gene profile of DNA:RNA hybrids at ORFs enriched for DNA:RNA hybrids under wild type conditions. (B–D) CHROMATRA plots of DNA:RNA hybrid distribution along genes sorted by their length (B), grouped into five transcriptional frequency categories as per [69]) (C) or grouped into four GC content categories (D). Genes were aligned by their TSSs. (E) The average DNA:RNA hybrid score at Ty1, Ty2, Ty3, Ty4 and Ty5 retrotransposons in the left panel shows higher enrichment at Ty1 and Ty2 retrotransposons. The average profile of DNA:RNA hybrids at all retrotransposons under wild type conditions is shown in the right panel.

Overall, our DRIP-chip profiles identified several previously reported DNA:RNA hybrid prone sites including the rDNA locus and telomeric repeat regions (**Figure 1**, **Supplementary Tables S1, S2**) [2], [29]–[31]. DNA:RNA hybrids were also observed at 1217 open reading frames (ORFs) (containing greater than 50% of probes above the threshold of 1.5 and found in both wild type replicates) (**Supplementary Table S3**). These were generally shorter in length than average (p = 4.29e^−58^), highly transcribed (Wilcoxon rank sum test p = 2.21e^−6^), and had higher GC content (p = 2.52e^−50^) (**Figure 2A**, **2B** and **2C**, **Supplementary Figure S2**). Importantly, despite the correlation between DNA:RNA hybrid association and transcriptional frequency, the wild type DRIP-chip profiles compared to the localization profile of the RNA polymerase II subunit Rpb3 revealed very low correlation (ρ = 0.0097; [45]). This suggests that the DRIP-chip method was not unduly biased towards the short DNA:RNA hybrids that could theoretically have been captured within active transcription bubbles. Importantly, because genes with high GC content also have high transcriptional frequencies (**Supplementary Figure S3**), it is not clear from our findings whether GC content or transcriptional frequency contributed more to DNA:RNA hybrid forming potential. Furthermore, we observe that DNA:RNA hybrid prone loci do not encode for mRNA transcripts with particularly long half-lives (**Supplementary Figure S2D**), suggesting that the act of transcription is vital to DNA:RNA hybrid formation and supporting the notion of co-transcriptional hybrid formation as the major source of endogenous DNA:RNA hybrids.

Our data also revealed DNA:RNA hybrids highly associated with Ty1 and Ty2 subclasses of retrotransposons (**Figure 2E**, **Supplementary Table S4**). Consistent with our findings at ORFs, the levels of DNA:RNA hybrids correspond well with the known levels of expression of these elements. In general, Ty1 which constitutes one of the most abundant transcripts in the cell has the highest levels of DNA:RNA hybrids. Ty3 and Ty4 that are only slightly expressed have much lower levels of hybrids, and the lone Ty5 retrotransposon which is transcriptionally silent is not enriched for DNA:RNA hybrids (**Figure 2E**) ([46]–[48]). In contrast to the trends observed with ORFs, GC content in retrotransposons is not highly correlated with the levels of expression, suggesting that expression is the main contributor to DNA:RNA hybrid formation. Specifically, Ty3 retrotransposons have the highest GC content but have only modest levels of expression and DNA:RNA hybrids.

### DNA:RNA hybrids are significantly correlated with genes associated with antisense transcripts

**Figure 3:**
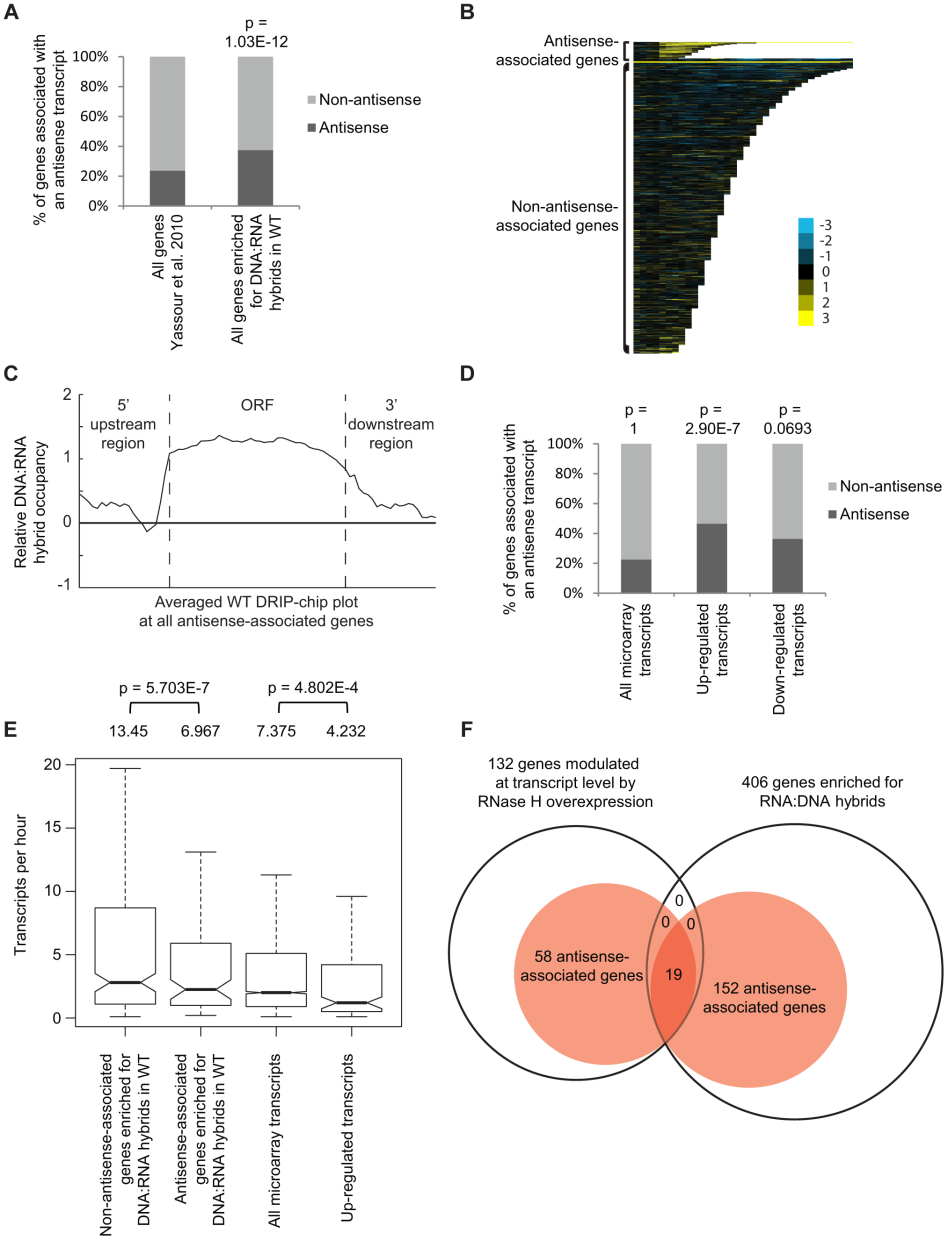
Genes associated with DNA:RNA hybrids were significantly associated with antisense transcripts. (A) Antisense association of DNA:RNA hybrid-enriched genes in wild type. The p-value indicates significant enrichment (Fisher's exact test) of antisense-associated genes among DNA:RNA hybrid-enriched genes compared to the Yassour et al. 2010 antisense-annotated dataset ([51]). (B) CHROMATRA plots of DNA:RNA hybrid distribution along genes sorted by their length and separated by whether they are antisense associated or not. Genes were aligned by their TSSs. (C) Average gene profile of DNA:RNA hybrids at genes associated with antisense transcripts. (D) Genes with increased mRNA levels upon RNase H overexpression were significantly associated with antisense transcripts compared to all transcripts represented by the microarray. (E) Antisense-associated DNA:RNA hybrid-enriched genes in wild type have lower transcription frequency compared to non-antisense-associated DNA:RNA hybrid-enriched genes. Genes up-regulated at the transcript level by RNase H overexpression have lower transcription frequency compared to all genes on the expression microarray. Intervals indicate range of the 95% of genes closest to the average in each sample. Averages stated above each bar. P values indicate significant decrease in transcriptional frequency (Wilcoxon rank sum test). (F) Overlap between DNA:RNA hybrid-enriched genes and RNase H-modulated transcripts sorted by antisense association according to the Yassour et al. 2010 database. For genes that are both hybrid-enriched and modulated at the transcript level by RNase H overexpression, the antisense association (100%) is significantly higher (Fisher's exact test p<2.2e^−16^) than those of the parent datasets (37.4% for DNA:RNA hybrid-enriched genes, 43.9% for RNase H-modulated genes).

Certain DNA:RNA hybrid enriched regions identified by our DRIP-chip analysis such as rDNA and retrotransposons are associated with antisense transcripts [49], [50]. Therefore, we checked if this was a common feature of DNA:RNA prone sites by comparing our list of DNA:RNA prone loci to a list of antisense-associated genes ([51]). Because the expression of antisense-associated transcripts may be highly dependent on environmental conditions, we based our analysis on a list of transcripts identified in S288c yeast grown to mid-log phase in rich media which most closely mirrors the growth conditions of our cultures analyzed by DRIP-chip ([51]). DNA:RNA hybrid enriched genes significantly overlapped with antisense-associated genes, suggesting that DNA:RNA hybrids may play a role in antisense transcript-mediated regulation of gene expression (Fisher's exact test p = 1.03e^−12^) (**Figure 3A**, **3B** and **3C**, **Supplementary Table S5**).

RNase H overexpression reduces detectable levels of DNA:RNA hybrids in cytological screens and suppresses genomic instability associated with R loop formation presumably through the degradation of DNA:RNA hybrids [7], [52], [53]. To test for a potential role of DNA:RNA hybrids in antisense-mediated gene regulation, we performed gene expression microarray analysis of an RNase H overexpression strain compared to an empty vector control (GEO GSE46652). This identified genes that had increased mRNA levels (upregulated n = 212) or decreased mRNA levels (downregulated n = 88) as a result of RNase H overexpression. A significant portion of the genes with increased mRNA levels were antisense-associated (Fisher exact test p = 2.9e^−7^) (**Figure 3D**, **Supplementary Table S5**) and tended to have high GC content, similar to DNA:RNA hybrid enriched genes in wild type (**Supplementary Figure S4**). However, the genes with increased mRNA levels under RNase H overexpression and the antisense-associated genes enriched for DNA:RNA hybrids in our DRIP experiment both tended towards lower transcriptional frequencies (**Figure 3E**). These findings suggest that antisense-associated DNA:RNA hybrids moderate the levels of gene expression. Indeed, genes that were both modulated by RNase H overexpression and enriched for DNA:RNA hybrids were all found to be antisense-associated (**Figure 3F**).

**Figure 4:**
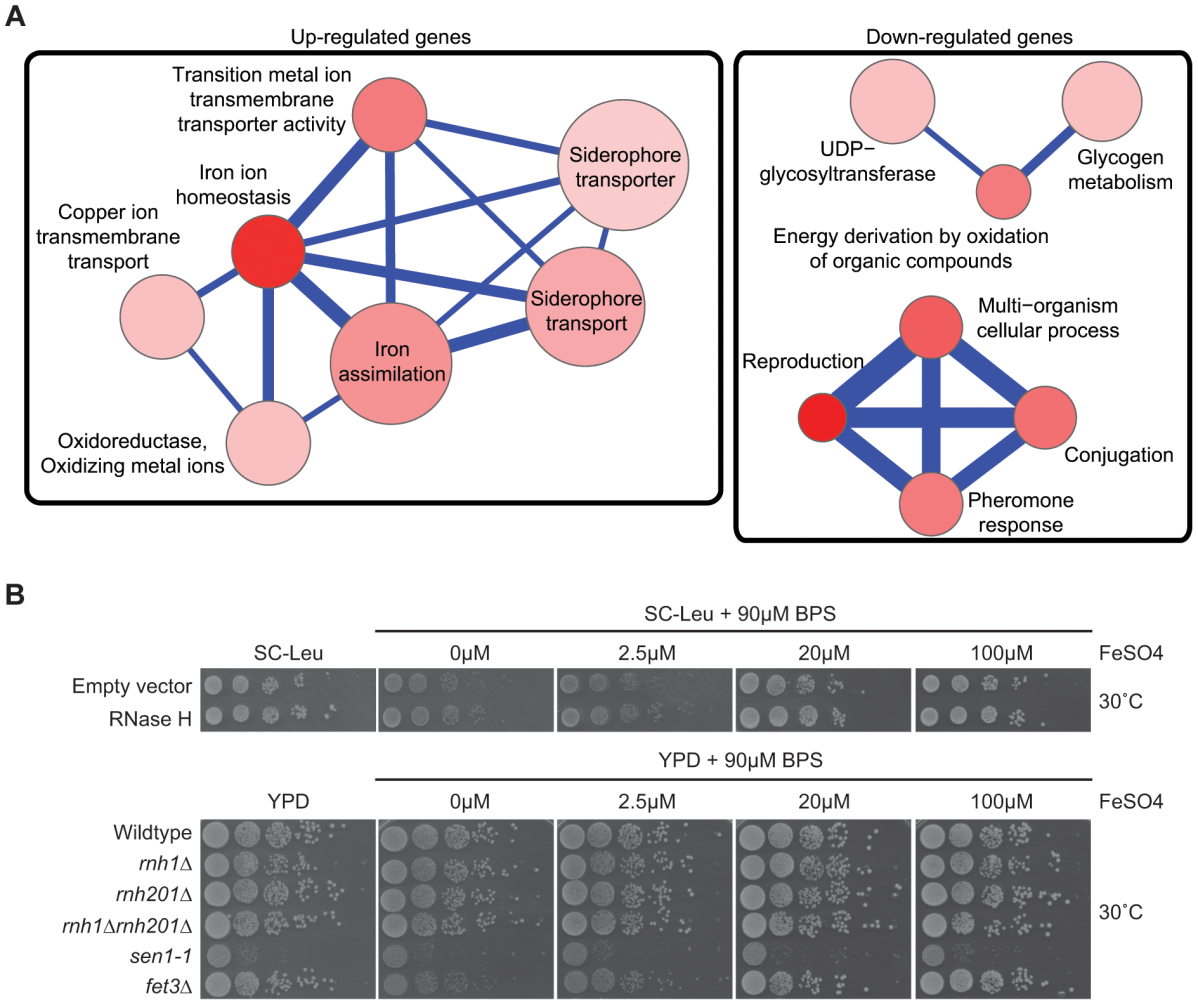
Pathways altered at the transcript level by RNase H overexpression. (A) Gene Ontology term network of genes with increased (left) or decreased (right) mRNA levels upon RNase H overexpression. Representative terms from **Supplementary Table S10** are shown. Node size indicates fold enrichment. Node color indicates the number of genes associated with each term (the darkest indicating the greatest number of genes associated). Edge thickness indicates the number of genes shared between terms. (B) 10-fold serial dilutions on BPS iron plates testing low iron concentration sensitivity of wild type versus DNA:RNA hybrid forming mutants reveals a lack of cellular iron requirement in RNase H mutant strains.

The mechanism underlying altered gene expression in cells overexpressing RNase H remains unclear. While the association with antisense transcription is compelling, alternative models exist. One possibility is that the stress of RNase H overexpression triggers gene expression programs that coincidentally are antisense regulated. We analyzed gene ontology (GO) terms enriched among genes whose expression was changed by RNase H overexpression. Consistent with previous work, genes for iron uptake and incorporation were strongly activated by RNase H overexpression (p = 2.21e^−12^) (**Figure 4A**, **Supplementary Table S6**) and several of these iron transport genes (i.e. *FRE4*, *FRE2*, *FRE3*, *FET3*, *FET4*) are antisense-associated ([51], [54]) suggesting that overexpression of RNase H activates transcription of these genes by perturbing antisense-mediated regulation. Alternatively, changes in RNase H levels may increase the cellular iron requirements since sensitivity to low iron concentration is associated with DNA damage and repair [55]. To test this alternative hypothesis, we tested the RNase H deletion and *sen1-1* mutants for sensitivity to low iron conditions compared to a *fet3Δ* positive control (**Figure 4B**). The *sen1-1* mutant, RNase H depletion or overexpression did not induce sensitivity to low iron ruling out the possibility that the transcriptional response in cells overexpressing RNase H was a result of cellular iron requirement. Collectively, our DRIP-chip and microarray analysis suggest that DNA:RNA hybrids may be an important player in antisense-mediated gene regulation.

### Cytological profiling of RNA processing mutants for R loop formation

**Figure 5:**
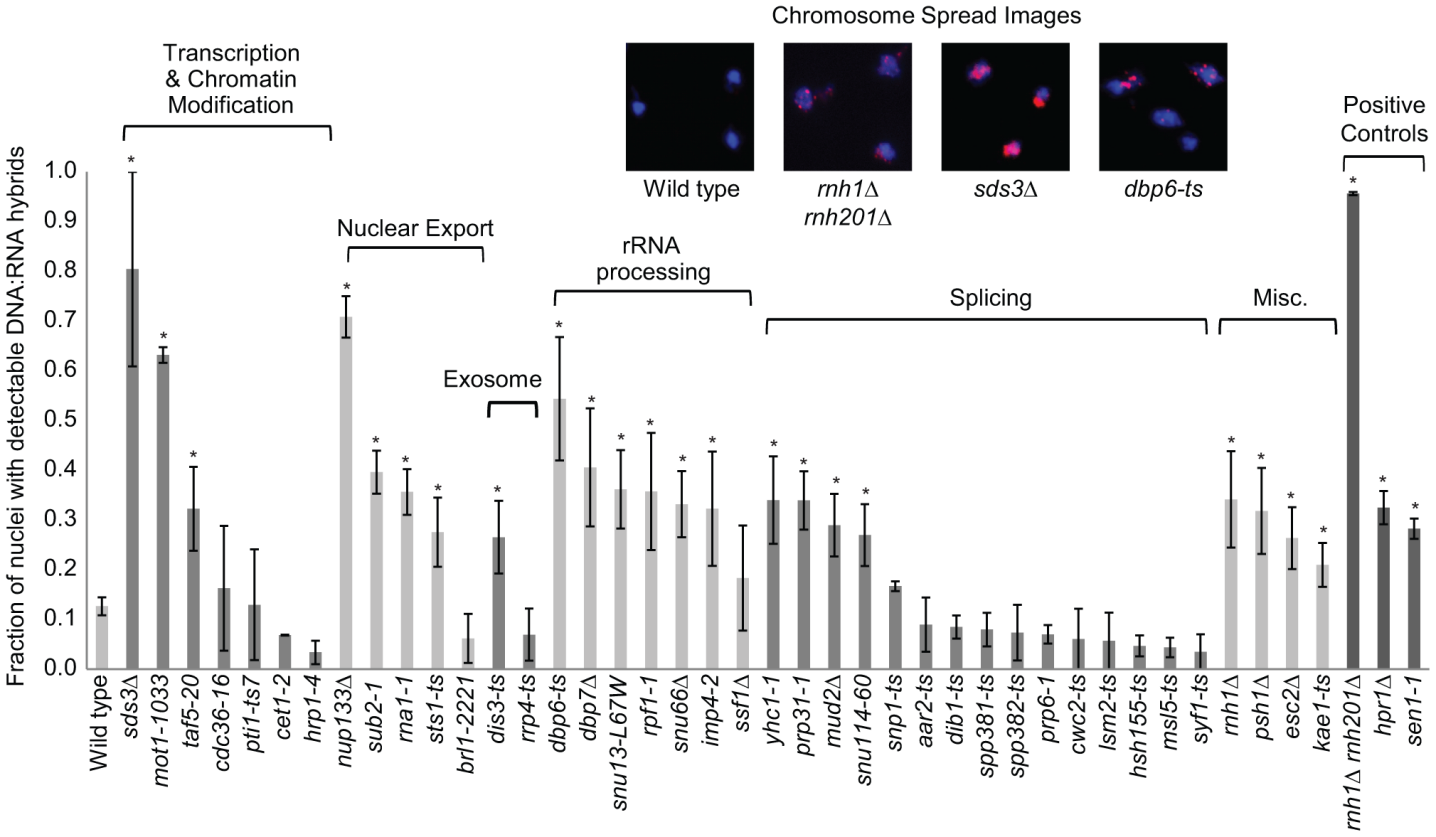
DNA:RNA hybrid cytological screen revealed high DNA:RNA hybrid levels in RNA processing and chromatin modification mutants. Asterisks indicate mutants with significantly increased levels of DNA:RNA hybrids compared to wild type (p<0.00024). Error bars indicate standard error of the mean. Representative chromosome spreads are shown: blue stain is DNA (DAPI) and the red foci are DNA:RNA hybrids.

Transcription-coupled DNA:RNA hybrids have been shown to accumulate in a diverse set of transcription and RNA processing mutants involved in a wide range of transcription related processes (**Table 1**). To gain a broader understanding of factors involved in R loop formation, we performed a cytological screen of RNA processing, transcription and chromatin modification mutants for DNA:RNA hybrids using the S9.6 antibody. Importantly, previous work in our lab has shown that all of the mutants screened exhibit chromosome instability (CIN), which would be consistent with increased hybrid formation [53]. Significantly elevated hybrid levels were found in 22 of the 40 mutants tested compared to wild type, including a *SUB2* mutant which has been previously linked to R loop formation (**Figure 5**, [4]). We also assayed some of the well-characterized R-loop forming mutants, RNase H, Sen1 and Hpr1, as positive controls for elevated DNA:RNA hybrid levels (**Figure 5**).

**Table 1:** List of yeast genes that affect DNA:RNA hybrid formation.

Yeast gene linked to DNA:RNA hybrid formation	Reference
Exosome and RNA degradation: *DIS3, RRP6, XRN1*	This study, [7], [22]
Helicase: *SEN1, SRS2*	[5], [9]
mRNA cleavage and polyadenylation: *CLP1, CFT2, FIP1, PCF11, RNA14, RNA15, TRF4*	[6], [22], [75]
mRNA export: *MEX67, MTR2, NAB2, NUP133, RNA1, SAC3, SRM1, SUB2, SUS1, THP1, YRA1*	This study, [6], [19], [22], [76], [77]
Other processes: *ESC2, KAE1, PSH1, STS1*	This study
RNA Polymerase II transcription and chromatin modification: *LEO1, MED12, MED13, MOT1, NPL3, RTT103, SDS3, SIN3, SPT2, TAF5*	This study, [7], [9], [23], [78]
RNase H: *RNH201, RNH1*	This study, [6], [7]
rRNA processing factors: *DBP6, DBP7, IMP4, RPF1, SNU13, SNU66*	This study
Splicing: *MUD2, SNU114, PRP31, YHC1, SNU13, SNU66*	This study
THO transcription elongation: *THO2, HPR1, MFT1, THP2*	[6], [18], [58]
Topoisomerase: *TOP1*	[2]

In our screen, we detected hybrids in mutants affecting several pathways linked to DNA:RNA hybrid formation such as transcription, nuclear export and the exosome (**Figure 5**, **Table 1**). Consistent with findings in metazoan cells, we also observed hybrid formation in some splicing mutants (**Figure 5**, **Table 1**; [56]). Several rRNA processing mutants were enriched for DNA:RNA hybrids (7 out of the 22 positive hits), likely due to DNA:RNA hybrid accumulation at rDNA genes, a sensitized hybrid formation site (**Figure 1**; [2]). It is possible that, as seen in mRNA cleavage and polyadenylation mutants, DNA:RNA hybrid formation may contribute to their CIN phenotypes [6]. Currently, there are 52 yeast genes whose disruptions have been found to lead to DNA:RNA hybrid accumulation, 21 of which were newly identified by our screen (**Table 1**). The success of this small-scale screen suggests that most RNA processing pathways suppress hybrid formation to some degree and that many DNA:RNA hybrid forming mutants remain undiscovered.

### DRIP-chip profiling of R loop forming mutants

**Figure 6:**
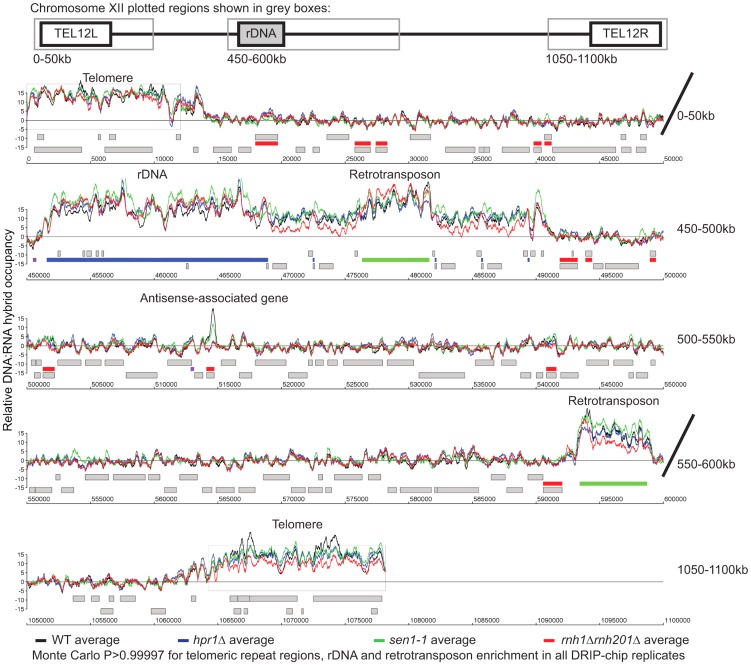
Genome-wide profiles of DNA:RNA hybrids in revealed similar enrichment of rDNA, retrotransposons and telomeres in wild type and mutants. DRIP-chip chromosome plot of DNA:RNA hybrids in wild type, *rnh1* ***Δ*** *rnh201* ***Δ***, *hpr1* ***Δ*** and *sen1-1* at chromosome XII. The average of two replicates per strain is shown. Bars indicate ORFs (grey), rDNA (purple), retrotransposons (green) or genes associated with an antisense transcript (red) [51], [54]). Grey boxes delineate telomeric repeat regions. Y-axis indicates relative occupancy of DNA:RNA hybrids. X-axis indicates chromosomal coordinates. P indicates probability of observing a number of enriched features below what was observed (P>0.99997).

**Figure 7:**
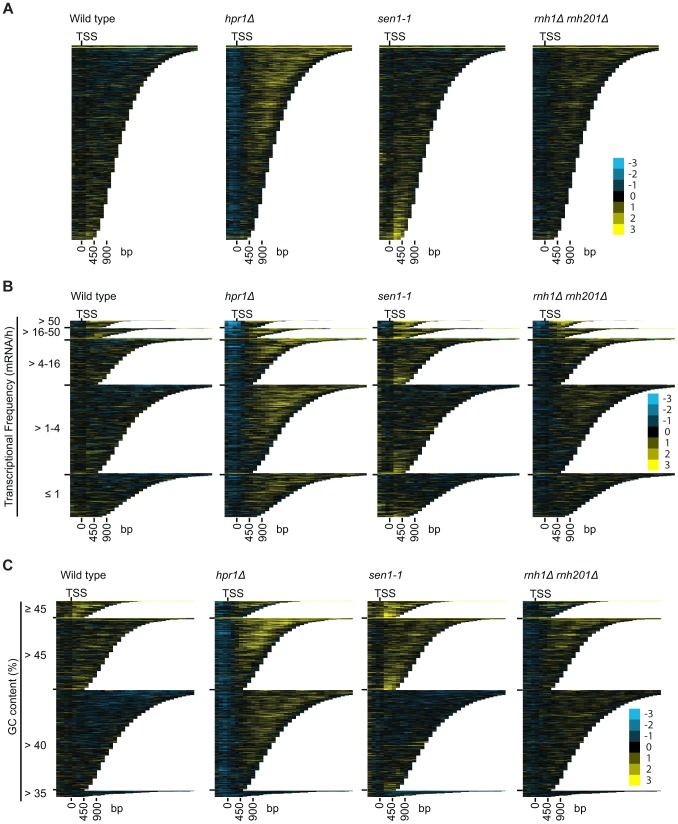
Mutant specific trends in protein-coding genes prone to DNA:RNA hybrid formation. (A–C) CHROMATRA plots of DNA:RNA hybrid distribution along genes sorted by their length (A) grouped into five transcriptional frequency categories as per [69] (B) or grouped into four GC content categories (C). Genes were aligned by their TSSs.

To better understand the mechanism by which cells regulate DNA:RNA hybrids, we performed DRIP-chip analysis of *rnh1Δrnh201Δ*, *hpr1*Δ, and *sen1-1* mutants in order to determine if these contribute differentially to the DNA:RNA hybrid genomic profile. The *rnh1Δrnh201Δ*, *hpr1*Δ, and *sen1-1* mutants are particularly interesting because they have well established roles in the regulation of transcription dependent DNA:RNA hybrid formation. Our DRIP-chip profiles revealed that, similar to wild type profiles, the mutant profiles were enriched for DNA:RNA hybrids at rDNA, telomeres, and retrotransposons (**Figure 6**, **Supplementary Tables S1, S2, S3**). The *rnh1Δrnh201Δ*, *hpr1*Δ, and *sen1-1* mutants also exhibited DNA:RNA hybrid enrichment in 1206, 1490 and 1424 ORFs respectively compared to the 1217 DNA:RNA hybrid enriched ORFs identified in wild type (**Supplementary Table S4**). Interestingly, in addition to the similarities described above, our profiles also identified differential effects of the mutants on the levels of DNA:RNA hybrids. In particular, we observed that deletion of *HPR1* resulted in higher levels of DNA:RNA hybrids along the length of most ORFs with a preference for longer genes compared to wild type (**Figure 7A**, **7B** and **7C**). This observation is consistent with Hpr1's role in bridging transcription elongation to mRNA export and its localization at actively transcribed genes ([4], [57]–[59]). In contrast, mutating *SEN1* resulted in higher levels of DNA:RNA hybrids at shorter genes (**Figure 7A** and **7B**), which is consistent with Sen1's role in transcription termination particularly for short protein-coding genes ([5], [60], [61]). The *rnh1Δrnh201Δ* mutant revealed higher levels of DNA:RNA hybrids at highly transcribed and longer genes (**Figure 7A** and **7B**) which is supported by a wealth of evidence of RNase H's role in suppressing R loops in long genes to prevent collisions between transcription and replication machineries ([8], [62]).

**Figure 8:**
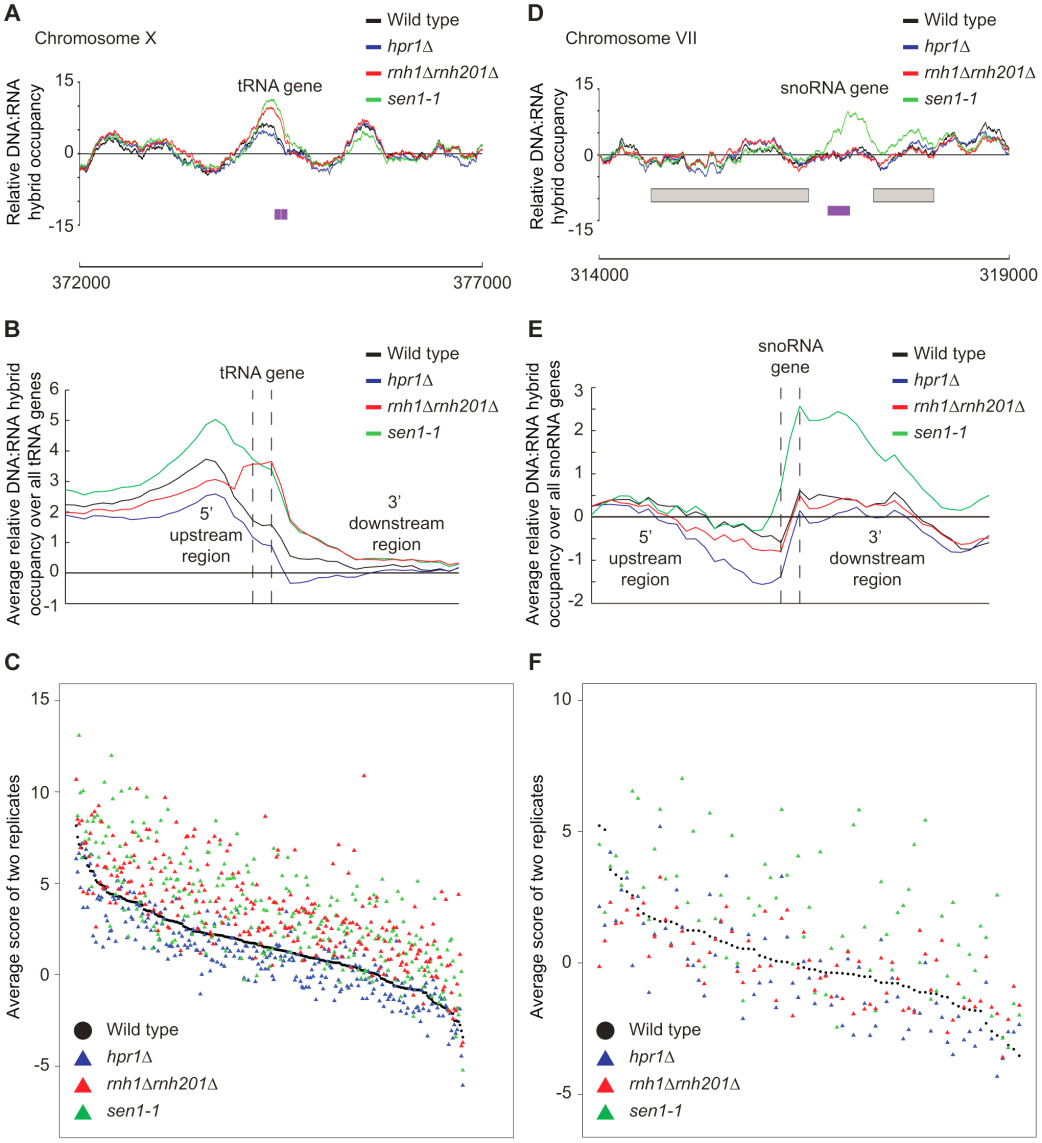
RNase H and Sen1 mutants displayed elevated levels of DNA:RNA hybrids at tRNA and snoRNA genes. (A) Sample plot of relative DNA:RNA hybrid occupancy at a tRNA gene on chromosome X. For A and D, Colored lines represent the average enrichment of the indicated strains. Purple bars indicate the tRNA or snoRNA genes respectively and gray boxes represent ORFs. (B) Average profile of DNA:RNA hybrids at all tRNAs. (C) Average DNA:RNA hybrid score at each tRNA. (D) Sample plot of relative DNA:RNA hybrid occupancy at a snoRNA gene on chromosome VII. (E) Average profile of DNA:RNA hybrids at all snoRNAs. (F) Average DNA:RNA hybrid score at each snoRNA. P indicates probability of observing a number of enriched features below what was observed (P>0.99997).

Further inspection of our profiles also revealed that *rnh1Δrnh201Δ* and *sen1-1* mutants but not the *hpr1*Δ mutant had increased DNA:RNA hybrids at tRNA genes (two tailed unpaired Wilcox test p = 1.56e^−19^ in the *rnh1Δrnh201Δ* mutant and 1.68e^−15^ in the *sen1-1* mutant) (**Figure 8A**, **8B** and **8C**, **Supplementary Table S7**) and this was confirmed by DRIP-quantitative PCR (qPCR) of two tRNA genes in wild type and *rnh1Δrnh201Δ* (**Supplementary Figure S5**). Because tRNAs are transcribed by RNA polymerase III, this observation indicates that Hpr1 is primarily involved in the regulation of RNA polymerase II specific DNA:RNA hybrids while RNase H and Sen1 have roles in a wider range of transcripts. Mutation of *SEN1* also led to increased levels DNA:RNA hybrids at snoRNA (two tailed unpaired Wilcox test p = 1.81e^−6^) (**Figure 8D**, **8E** and **8F**, **Supplementary Table S8**) consistent with its role in 3′ end processing of snoRNAs ([63]).

## Discussion

### The genomic profile of DNA:RNA hybrids

Identifying the landscape of genomic loci predisposed to DNA:RNA hybrids is of fundamental importance to delineating mechanisms of hybrid formation and the contributions of various cellular pathways. Although our profiles depend on the specificity of the anti-DNA:RNA hybrid S9.6 monoclonal antibody, this aspect has been well characterized [44] and several of our observations are consistent with what has been reported in the literature. Locus specific tests showed that DNA:RNA hybrids occur more frequently at genes with high transcriptional frequency and GC content [4], [5], [18]. Moreover, in *rnh201*Δ cells, there is an inverse relationship between GC content and gene expression levels, suggesting that DNA:RNA hybrids accumulate at regions of high GC content and block transcription in the absence of RNase H [64]. Our work extends the knowledge of DNA:RNA hybrids from a few locus-specific observations to show that, in wild type, there are potentially hundreds of hybrid prone genes that tend to be shorter in length, frequently transcribed and high in GC content [2], [4], [56]. The latter is consistent with recent studies in human cells that demonstrated that genomic regions with high GC skew are prone to R loop formation, which plays a regulatory role in DNA methylation [26], [27]. However, while we determined the relationship between GC content and DNA:RNA hybrid formation, we were unable to do the same analysis for GC skew, likely due to the low level of GC skew and lack of DNA methylation in *Saccharomyces*. This is unsurprising since the best characterized functional element associated with GC skew, CpG island promoters [26], , are not found in yeast. Importantly, our findings at retrotransposons support the notion that expression levels and not GC content contribute more to DNA:RNA hybrid forming potential. Additionally, DRIP-chip analysis of wild type cells identified hybrid enrichment at rDNA, retrotransposons, and telomeric regions. Along with previous studies, our DRIP-chip analysis confirms that rDNA is a hybrid prone genomic site and suggests that many factors of rRNA processing and ribosome assembly suppress potentially damaging rDNA:rRNA hybrid formation [2], [7]. The presence of TERRA-DNA hybrids at telomeres is supported by our observation of significant hybrid signal at telomeric repeat regions across all DRIP-chip experiments.

### Antisense association of DNA:RNA hybrids

The DRIP-chip dataset is a resource for future studies seeking to elucidate the localization of DNA:RNA hybrids across antisense-associated regions and the impact of DNA:RNA hybrid removal on genome-wide transcription. We observed that genes associated with antisense transcripts were significantly enriched for DNA:RNA hybrids and modulated at the transcript level by RNase H overexpression. Antisense regulation has been reported at mammalian rDNA and yeast Ty1 retrotransposons, loci that were also enriched for DNA:RNA hybrids in our DRIP-chip [49], [50]. The role of DNA:RNA hybrids and RNase H in antisense regulation is currently unclear. However, there are several non-exclusive models of antisense gene regulation. One model proposes that the physical presence of the antisense transcripts is crucial to antisense gene regulation. For instance, *trans*-acting antisense transcripts have been shown to control Ty1 retrotransposon transcription, reverse transcription and retrotransposition [65]. Another study has further shown that *trans*-acting antisense transcripts that only overlap with the sense strand promoter can block sense transcription, potentially by hybridizing with the non-template DNA strand [33]. These suggest that antisense transcription in *cis* is not necessary as long as the antisense transcript is present. It is possible that DNA:RNA hybrids may be formed by the antisense or the sense transcript with genomic DNA. Moreover, DNA:RNA hybrids may play a functional role in antisense transcription regulation as shown by antisense-associated genes both enriched for DNA:RNA hybrids and affected transcriptionally by RNase H overexpression. Experiments comparing the ratio of antisense versus sense transcripts and determining the amount of DNA:RNA hybrid formation by either transcript under conditions known to regulate the particular gene will further elucidate the role of RNase H and DNA:RNA hybrids in antisense regulation.

### DRIP-chip analysis of hybrid-resolving mutants

Our investigation of mutant-specific DNA:RNA hybrid formation sites is consistent with the existing literature on Hpr1, Sen1 and RNase H. Significantly, the *hpr1*Δ and *rnh1Δrnh201Δ* mutants exhibited increased DNA:RNA hybrid levels along the length of long genes, while the *sen1-1* mutant exhibited increased DNA:RNA hybrid levels along the length of short genes (**Figure 7A**). This coheres with Hpr1's function in transcription elongation and mRNA export, and RNase H's role in preventing transcription apparatus and replication fork collisions, which carry greater consequence for long genes ([4], [57]–[59], [62]). In contrast, Sen1 is particularly important for transcription termination at short genes ([61]).

In addition, the RNase H deletion and *sen1-1* mutants had increased hybrids at tRNA genes, suggesting that they are both required to prevent tRNA:DNA hybrid accumulation. Interestingly, a recent study found that the mRNA levels of genes encoding RNA polymerase III and proteins that modify tRNA are increased in an *rnh1Δrnh201Δ* mutant [64], which may be in response to a lack of properly processed tRNA transcripts. The finding that both tRNA and snoRNA genes were enriched for hybrids in *sen1-1* highlights the role of Sen1 in RNA polymerase I, II and III transcription termination and transcript maturation [60], [63], [66]. More broadly, our data and the literature support the notion that transcripts from RNA polymerases I, II and III can be subject to DNA:RNA hybrid formation especially in RNA processing mutant backgrounds.

### Perspective

Factors regulating ectopic, genome destabilizing DNA:RNA hybrids are best characterized in yeast, although less is known about the functions of native R loop structures. The genome-wide maps of DNA:RNA hybrids presented here recapitulate the known sites of hybrid formation but also add important new insights to potential functions of R loops. Most importantly, we demonstrate the usefulness of DRIP profiling for detecting biologically meaningful differences in mutant strains. Therefore, DRIP profiling of yeast genomes in various mutant backgrounds will be key to understanding the causes and consequences of inappropriate R loop formation and how these are modulated by other cellular pathways.

## Methods

### Strains and plasmids

All strains are listed in **Supplementary Table S9**. For RNase H overexpression experiments, recombinant human RNase H1 was expressed from plasmid p425-GPD-RNase H1 (2μ, LEU2, GPDpr-RNase H1) and compared to an empty control plasmid p425-GPD (2μ, LEU2, GPDpr) [7].

### DRIP-chip and qPCR

Briefly, cells were grown overnight, diluted to 0.15 OD_600_ and grown to 0.7 OD_600_. Crosslinking was done with 1% formaldehyde for 20 minutes. Chromatin was purified as described previously [67] and sonicated to yield approximately 500 bp fragments. 40 µg of the anti-DNA:RNA hybrid monoclonal mouse antibody S9.6 (gift from Stephen Leppla) was coupled to 60 µL of protein A magnetic beads (Invitrogen). For ChIP-qPCR, crosslinking reversal and DNA purification were followed by qPCR analysis of the immunoprecipitated and input DNA. DNA was analyzed using a Rotor-Gene 600 (Corbett Research) and PerfeCTa SYBR green FastMix (Quanta Biosciences). Samples were analyzed in triplicate on three independent DRIP samples for wild type and *rnh1*Δ*rnh201*Δ. Primers are listed in **Supplementary Table S11**.

For DRIP-chip, precipitated DNA was amplified via two rounds of T7 RNA polymerase amplification ([68]), biotin labeled and hybridized to Affymetrix 1.0R *S. cerevisiae* microarrays. Samples were normalized to a no antibody control sample (mock) using the rMAT software and relative occupancy scores were calculated for all probes using a 300 bp sliding window. All profiles were generated in duplicate and replicates were quantile normalized and averaged. Spearman correlation scores between replicates are listed in **Supplementary Table S10**. Coordinates of enriched regions are available in **Dataset S1/S2/S3/S4/S5/S6/S7/S8**. DRIP-chip data is available at ArrayExpress E-MTAB-2388.

### DRIP-chip analysis

Enriched features had at least 50% of the probes contained in the feature above the threshold of 1.5. Only features enriched in both replicates were reported. Transcriptional frequency [69], GC content ([70]) and gene length were compared using the Wilcoxon rank sum test. Antisense association was analyzed by the Fisher's exact test using R. Statistical analysis of genomic feature enrichment was performed using a Monte Carlo simulation, which randomly generates start positions for the particular set of features and calculates the proportion of that feature that would be enriched in a given DRIP-chip profile if the feature were distributed at random [67]. 500 simulations were run per feature for each DRIP-chip replicate to obtain mean and standard deviation values. These values were used to calculate the cumulative probability (P) on a normal distribution of seeing a score lower than the observed value by chance.

### DRIP-chip visualization

CHROMATRA plots were generated as described previously ([71]). Relative occupancy scores for each transcript were binned into segments of 150 bp. Transcripts were sorted by their length, transcriptional frequency or GC content and aligned by their Transcription Start Sites (TSS). For transcriptional frequency transcripts were grouped into five classes according to their transcriptional frequency described by Holstege *et al* 1998. For GC content transcripts were grouped into four classes according to their GC content obtained from BioMart ([70]). Average gene, tRNA or snoRNA profiles were generated by averaging all the probes that were encompassed by the features of interest. For averaging ORFs, corresponding probes were split into 40 bins while 1500 bp of UTRs and their probes were split into 20 bins. For smaller features like tRNAs and snoRNAs corresponding probes were split into only 3 bins. Average enrichment scores were calculated using in house scripts that average the score of all the probes encompassed by the feature.

### Gene expression microarray

Gene expression microarray data is available at GEO GSE46652. Strains harboring the RNase H1 over-expression plasmid or empty vector were grown in SC-Leucine at 30°C. All profiles were generated in duplicate. Total RNA was isolated from 1 OD_600_ of yeast cells using a RiboPure Yeast kit (A&B Applied Biosystems), amplified, labeled, fragmented using a Message-Amp III RNA Amplification Kit (A&B Applied Biosystems) and hybridized to a GeneChIP Yeast Genome 2.0 microarray using the GeneChip Hybridization, Wash, and Stain Kit (Affymetrix). Arrays were scanned by the Gene Chip Scanner 3000 7G and expression data was extracted using Expression Console Software (Affymetrix) with the MAS5.0 statistical algorithm. All arrays were scaled to a median target intensity of 500. A minimum cut off of p-value of 0.05 and signal strength of 100 across all samples were implemented and only transcripts that had over a 2-fold change in the RNase H over-expression strain compared to wild type were considered significant. The correlation between duplicate biological samples was: control (r = 0.9955), RNase H over-expression (r = 0.9719). For statistical analysis, GC content, transcription frequencies and antisense association were analyzed as for DRIP-chip analysis.

### Yeast chromosome spreads

Cells were grown to mid-log phase in YEPD rich media at 30°C and washed in spheroplasting solution (1.2 M sorbitol, 0.1 M potassium phosphate, 0.5 M MgCl_2_, pH 7) and digested in spheroplasting solution with 10 mM DTT and 150 µg/mL Zymolase 20T at 37°C for 20 minutes similar to previously described ([72]). The digestion was halted by addition of ice-cold stop solution (0.1 M MES, 1 M sorbital, 1 mM EDTA, 0.5 mM MgCl_2_, pH 6.4) and spheroplasts were lysed with 1% vol/vol Lipsol and fixed on slides using 4% wt/vol paraformaldehyde/3.4% wt/vol sucrose ([73]). Chromosome spread slides were incubated with the mouse monoclonal antibody S9.6 (1 µg/mL in blocking buffer of 5% BSA, 0.2% milk and 1× PBS). The slides were further incubated with a secondary Cy3-conjugated goat anti-mouse antibody (Jackson Laboratories, #115-165-003, diluted 1∶1000 in blocking buffer). For each replicate, at least 100 nuclei were visualized and manually counted to obtain the fraction with detectable DNA:RNA hybrids. Each mutant was assayed in triplicate. Mutants were compared to wild type by the Fisher's exact test. To correct for multiple hypothesis testing, we implemented a cut off of p<0.01 divided by the total number of mutants compared to wild type, meaning mutants with p<0.00024 were considered significantly different from wild type.

### BPS sensitivity assay

10-fold serial dilutions of each strain was spotted on 90 µM BPS plates with FeSO_4_ concentrations of 0, 2.5, 20 or 100 µM and grown at 30°C for 3 days [55].

A summary of this paper was presented at the 26^th^ International Conference on Yeast Genetics and Molecular Biology, August 2013 [74].

## Supporting Information

Dataset S1 Wild type replicate 1 enriched region coordinates.
(XLSX)

Dataset S2 Wild type replicate 2 enriched region coordinates.
(XLSX)

Dataset S3 *sen1-1* replicate 1 enriched region coordinates.
(XLSX)

Dataset S4 *sen1-1* replicate 2 enriched region coordinates.
(XLSX)

Dataset S5 *hpr1Δ* replicate 1 enriched region coordinates.
(XLSX)

Dataset S6 *hpr1Δ* replicate 2 enriched region coordinates.
(XLSX)

Dataset S7 *rnh1Δ rnh201Δ* replicate 1 enriched region coordinates.
(XLSX)

Dataset S8 *rnh1Δ rnh201Δ* replicate 2 enriched region coordinates.
(XLSX)

Figure S1 Spearman correlation scatter plot of wild type replicates.
(EPS)

Figure S2 Box plots comparing the distribution of (A) gene length, (B) transcription frequency, (C) GC content, and (D) mRNA transcript half-life of ORFs enriched for DNA:RNA hybrids versus ORFs not enriched for DNA:RNA hybrids. The p values calculated by the Wilcoxon rank sum test are shown.
(EPS)

Figure S3 (A) Distribution of % GC content of all ORFs sorted by transcriptional frequency. (B) Distribution of % GC content of ORFs enriched for DNA:RNA hybrids in WT sorted by transcriptional frequency. Intervals indicate 95% confidence intervals.
(EPS)

Figure S4 Distribution of % GC content of all genes represented on the expression microarray (n = 5657), and transcripts up- (n = 212) or down-regulated (n = 88) by RNase H overexpression. Intervals indicate 95% confidence intervals. Averages are stated above each sample. The p-value indicates a significant increase in GC content of upregulated genes compared to all microarray transcripts (Wilcoxon rank sum test).
(EPS)

Figure S5 Relative quantities of (A) *SUF2* tRNA gene and (B) *tV(UAC)D* tRNA gene detected in WT or *rnh1Δrnh201Δ* as detected by DRIP-quantitative PCR (qPCR). Error bars indicate standard deviation.
(EPS)

Table S1 List of rDNA enriched for RNA:DNA hybrids in wild type, *rnh1Δrnh201Δ*, *hpr1Δ* and *sen1-1*.
(XLSX)

Table S2 List of telomeric repeat regions enriched for RNA:DNA hybrids in wild type, *rnh1Δrnh201Δ*, *hpr1Δ* and *sen1-1*.
(XLSX)

Table S3 List of ORFs enriched for RNA:DNA hybrids in wild type, *rnh1Δrnh201Δ*, *hpr1Δ* and *sen1-1*.
(XLSX)

Table S4 List of retrotransposons enriched for RNA:DNA hybrids in wild type, *rnh1Δrnh201Δ*, *hpr1Δ* and *sen1-1*.
(XLSX)

Table S5 Lists of open reading frames (ORFs) and antisense-associated ORFs enriched for RNA:DNA hybrids in wild type or modulated at the transcript level by RNase H overexpression.
(XLSX)

Table S6 GO function sorting of genes modulated at the transcript level by RNase H overexpression.
(XLSX)

Table S7 List of tRNA genes enriched for RNA:DNA hybrids in wild type, *rnh1Δrnh201Δ*, *hpr1Δ* and *sen1-1*.
(XLSX)

Table S8 List of snoRNA genes enriched for RNA:DNA hybrids in wild type, *rnh1Δrnh201Δ*, *hpr1Δ* and *sen1-1*.
(XLSX)

Table S9 Strains used in this study.
(XLSX)

Table S10 Spearman correlation scores.
(XLSX)

Table S11 DRIP-qPCR primers.
(XLSX)

## References

[1] Chernikova SB, Razorenova OV, Higgins JP, Sishc BJ, Nicolau M, et al (2012) Deficiency in mammalian histone H2B ubiquitin ligase Bre1 (Rnf20/Rnf40) leads to replication stress and chromosomal instability. Cancer Res 72: 2111–2119. 22354749 10.1158/0008-5472.CAN-11-2209 PMC3328627

[2] El Hage A, French SL, Beyer AL, Tollervey D (2010) Loss of topoisomerase I leads to R-loop-mediated transcriptional blocks during ribosomal RNA synthesis. Genes Dev 24: 1546–1558. 20634320 10.1101/gad.573310 PMC2904944

[3] Gan W, Guan Z, Liu J, Gui T, Shen K, et al (2011) R-loop-mediated genomic instability is caused by impairment of replication fork progression. Genes Dev 25: 2041–2056. 21979917 10.1101/gad.17010011 PMC3197203

[4] Gomez-Gonzalez B, Garcia-Rubio M, Bermejo R, Gaillard H, Shirahige K, et al (2011) Genome-wide function of THO/TREX in active genes prevents R-loop-dependent replication obstacles. EMBO J 30: 3106–3119. 21701562 10.1038/emboj.2011.206 PMC3160181

[5] Mischo HE, Gomez-Gonzalez B, Grzechnik P, Rondon AG, Wei W, et al (2011) Yeast Sen1 helicase protects the genome from transcription-associated instability. Mol Cell 41: 21–32. 21211720 10.1016/j.molcel.2010.12.007 PMC3314950

[6] Stirling PC, Chan YA, Minaker SW, Aristizabal MJ, Barrett I, et al (2012) R-loop-mediated genome instability in mRNA cleavage and polyadenylation mutants. Genes Dev 26: 163–175. 22279048 10.1101/gad.179721.111 PMC3273840

[7] Wahba L, Amon JD, Koshland D, Vuica-Ross M (2011) RNase H and multiple RNA biogenesis factors cooperate to prevent RNA:DNA hybrids from generating genome instability. Mol Cell 44: 978–988. 22195970 10.1016/j.molcel.2011.10.017 PMC3271842

[8] Aguilera A, Garcia-Muse T (2012) R loops: From transcription byproducts to threats to genome stability. Mol Cell 46: 115–124. 22541554 10.1016/j.molcel.2012.04.009

[9] Wahba L, Gore SK, Koshland D (2013) The homologous recombination machinery modulates the formation of RNA-DNA hybrids and associated chromosome instability. Elife 2: e00505. 23795288 10.7554/eLife.00505 PMC3679537

[10] Castellano-Pozo M, Garcia-Muse T, Aguilera A (2012) R-loops cause replication impairment and genome instability during meiosis. EMBO Rep 13: 923–929. 22878416 10.1038/embor.2012.119 PMC3463965

[11] Dominguez-Sanchez MS, Barroso S, Gomez-Gonzalez B, Luna R, Aguilera A (2011) Genome instability and transcription elongation impairment in human cells depleted of THO/TREX. PLoS Genet 7: e1002386. 22144908 10.1371/journal.pgen.1002386 PMC3228816

[12] Hanahan D, Weinberg RA (2011) Hallmarks of cancer: The next generation. Cell 144: 646–674. 21376230 10.1016/j.cell.2011.02.013

[13] Crow YJ, Leitch A, Hayward BE, Garner A, Parmar R, et al (2006) Mutations in genes encoding ribonuclease H2 subunits cause aicardi-goutieres syndrome and mimic congenital viral brain infection. Nat Genet 38: 910–916. 16845400 10.1038/ng1842

[14] Garraway LA, Lander ES (2013) Lessons from the cancer genome. Cell 153: 17–37. 23540688 10.1016/j.cell.2013.03.002

[15] Papaemmanuil E, Cazzola M, Boultwood J, Malcovati L, Vyas P, et al (2011) Somatic SF3B1 mutation in myelodysplasia with ring sideroblasts. N Engl J Med 365: 1384–1395. 21995386 10.1056/NEJMoa1103283 PMC3322589

[16] Suraweera A, Lim Y, Woods R, Birrell GW, Nasim T, et al (2009) Functional role for senataxin, defective in ataxia oculomotor apraxia type 2, in transcriptional regulation. Hum Mol Genet 18: 3384–3396. 19515850 10.1093/hmg/ddp278

[17] Wang L, Lawrence MS, Wan Y, Stojanov P, Sougnez C, et al (2011) SF3B1 and other novel cancer genes in chronic lymphocytic leukemia. N Engl J Med 365: 2497–2506. 22150006 10.1056/NEJMoa1109016 PMC3685413

[18] Chavez S, Garcia-Rubio M, Prado F, Aguilera A (2001) Hpr1 is preferentially required for transcription of either long or G+C-rich DNA sequences in saccharomyces cerevisiae. Mol Cell Biol 21: 7054–7064. 11564888 10.1128/MCB.21.20.7054-7064.2001 PMC99881

[19] Jimeno S, Rondon AG, Luna R, Aguilera A (2002) The yeast THO complex and mRNA export factors link RNA metabolism with transcription and genome instability. EMBO J 21: 3526–3535. 12093753 10.1093/emboj/cdf335 PMC126085

[20] Alzu A, Bermejo R, Begnis M, Lucca C, Piccini D, et al (2012) Senataxin associates with replication forks to protect fork integrity across RNA-polymerase-II-transcribed genes. Cell 151: 835–846. 23141540 10.1016/j.cell.2012.09.041 PMC3494831

[21] Leela JK, Syeda AH, Anupama K, Gowrishankar J (2013) Rho-dependent transcription termination is essential to prevent excessive genome-wide R-loops in escherichia coli. Proc Natl Acad Sci U S A 110: 258–263. 23251031 10.1073/pnas.1213123110 PMC3538224

[22] Luna R, Jimeno S, Marin M, Huertas P, Garcia-Rubio M, et al (2005) Interdependence between transcription and mRNP processing and export, and its impact on genetic stability. Mol Cell 18: 711–722. 15949445 10.1016/j.molcel.2005.05.001

[23] Sikdar N, Banerjee S, Zhang H, Smith S, Myung K (2008) Spt2p defines a new transcription-dependent gross chromosomal rearrangement pathway. PLoS Genet 4: e1000290. 19057669 10.1371/journal.pgen.1000290 PMC2585797

[24] Wahba L, Koshland D (2013) The rs of biology: R-loops and the regulation of regulators. Mol Cell 50: 611–612. 23746348 10.1016/j.molcel.2013.05.024

[25] Chaudhuri J, Tian M, Khuong C, Chua K, Pinaud E, et al (2003) Transcription-targeted DNA deamination by the AID antibody diversification enzyme. Nature 422: 726–730. 12692563 10.1038/nature01574

[26] Ginno PA, Lim YW, Lott PL, Korf I, Chedin F (2013) GC skew at the 5′ and 3′ ends of human genes links R-loop formation to epigenetic regulation and transcription termination. Genome Res 23: 1590–1600. 23868195 10.1101/gr.158436.113 PMC3787257

[27] Ginno PA, Lott PL, Christensen HC, Korf I, Chedin F (2012) R-loop formation is a distinctive characteristic of unmethylated human CpG island promoters. Mol Cell 45: 814–825. 22387027 10.1016/j.molcel.2012.01.017 PMC3319272

[28] Skourti-Stathaki K, Proudfoot NJ, Gromak N (2011) Human senataxin resolves RNA/DNA hybrids formed at transcriptional pause sites to promote Xrn2-dependent termination. Mol Cell 42: 794–805. 21700224 10.1016/j.molcel.2011.04.026 PMC3145960

[29] Balk B, Maicher A, Dees M, Klermund J, Luke-Glaser S, et al (2013) Telomeric RNA-DNA hybrids affect telomere-length dynamics and senescence. Nat Struct Mol Biol 20: 1199–1205. 24013207 10.1038/nsmb.2662

[30] Luke B, Panza A, Redon S, Iglesias N, Li Z, et al (2008) The Rat1p 5′ to 3′ exonuclease degrades telomeric repeat-containing RNA and promotes telomere elongation in saccharomyces cerevisiae. Mol Cell 32: 465–477. 19026778 10.1016/j.molcel.2008.10.019

[31] Pfeiffer V, Crittin J, Grolimund L, Lingner J (2013) The THO complex component Thp2 counteracts telomeric R-loops and telomere shortening. EMBO J 32 (21) 2861–71. 24084588 10.1038/emboj.2013.217 PMC3817467

[32] Faghihi MA, Wahlestedt C (2009) Regulatory roles of natural antisense transcripts. Nat Rev Mol Cell Biol 10: 637–643. 19638999 10.1038/nrm2738 PMC2850559

[33] Camblong J, Beyrouthy N, Guffanti E, Schlaepfer G, Steinmetz LM, et al (2009) Trans-acting antisense RNAs mediate transcriptional gene cosuppression in S. cerevisiae. Genes Dev 23: 1534–1545. 19571181 10.1101/gad.522509 PMC2704465

[34] Castelnuovo M, Rahman S, Guffanti E, Infantino V, Stutz F, et al (2013) Bimodal expression of PHO84 is modulated by early termination of antisense transcription. Nat Struct Mol Biol 20: 851–858. 23770821 10.1038/nsmb.2598 PMC4972572

[35] Hobson DJ, Wei W, Steinmetz LM, Svejstrup JQ (2012) RNA polymerase II collision interrupts convergent transcription. Mol Cell 48: 365–374. 23041286 10.1016/j.molcel.2012.08.027 PMC3504299

[36] Kanhere A, Viiri K, Araujo CC, Rasaiyaah J, Bouwman RD, et al (2010) Short RNAs are transcribed from repressed polycomb target genes and interact with polycomb repressive complex-2. Mol Cell 38: 675–688. 20542000 10.1016/j.molcel.2010.03.019 PMC2886029

[37] Margaritis T, Oreal V, Brabers N, Maestroni L, Vitaliano-Prunier A, et al (2012) Two distinct repressive mechanisms for histone 3 lysine 4 methylation through promoting 3′-end antisense transcription. PLoS Genet 8: e1002952. 23028359 10.1371/journal.pgen.1002952 PMC3447963

[38] Marinello J, Chillemi G, Bueno S, Manzo SG, Capranico G (2013) Antisense transcripts enhanced by camptothecin at divergent CpG-island promoters associated with bursts of topoisomerase I-DNA cleavage complex and R-loop formation. Nucleic Acids Res 41 (22) 10110–23. 23999093 10.1093/nar/gkt778 PMC3905886

[39] van Dijk EL, Chen CL, d'Aubenton-Carafa Y, Gourvennec S, Kwapisz M, et al (2011) XUTs are a class of Xrn1-sensitive antisense regulatory non-coding RNA in yeast. Nature 475: 114–117. 21697827 10.1038/nature10118

[40] Wang X, Arai S, Song X, Reichart D, Du K, et al (2008) Induced ncRNAs allosterically modify RNA-binding proteins in cis to inhibit transcription. Nature 454: 126–130. 18509338 10.1038/nature06992 PMC2823488

[41] Sun Q, Csorba T, Skourti-Stathaki K, Proudfoot NJ, Dean C (2013) R-loop stabilization represses antisense transcription at the arabidopsis FLC locus. Science 340: 619–621. 23641115 10.1126/science.1234848 PMC5144995

[42] Powell WT, Coulson RL, Gonzales ML, Crary FK, Wong SS, et al (2013) R-loop formation at Snord116 mediates topotecan inhibition of Ube3a-antisense and allele-specific chromatin decondensation. Proc Natl Acad Sci U S A 110: 13938–13943. 23918391 10.1073/pnas.1305426110 PMC3752217

[43] Hu Z, Zhang A, Storz G, Gottesman S, Leppla SH (2006) An antibody-based microarray assay for small RNA detection. Nucleic Acids Res 34: e52. 16614443 10.1093/nar/gkl142 PMC1435976

[44] Boguslawski SJ, Smith DE, Michalak MA, Mickelson KE, Yehle CO, et al (1986) Characterization of monoclonal antibody to DNA.RNA and its application to immunodetection of hybrids. J Immunol Methods 89: 123–130. 2422282 10.1016/0022-1759(86)90040-2

[45] Aristizabal MJ, Negri GL, Benschop JJ, Holstege FC, Krogan NJ, et al (2013) High-throughput genetic and gene expression analysis of the RNAPII-CTD reveals unexpected connections to SRB10/CDK8. PLoS Genet 9: e1003758. 24009531 10.1371/journal.pgen.1003758 PMC3757075

[46] Clark DJ, Bilanchone VW, Haywood LJ, Dildine SL, Sandmeyer SB (1988) A yeast sigma composite element, TY3, has properties of a retrotransposon. J Biol Chem 263: 1413–1423. 2447089

[47] Hug AM, Feldmann H (1996) Yeast retrotransposon Ty4: The majority of the rare transcripts lack a U3-R sequence. Nucleic Acids Res 24: 2338–2346. 8710505 10.1093/nar/24.12.2338 PMC145937

[48] Ke N, Irwin PA, Voytas DF (1997) The pheromone response pathway activates transcription of Ty5 retrotransposons located within silent chromatin of saccharomyces cerevisiae. EMBO J 16: 6272–6280. 9321406 10.1093/emboj/16.20.6272 PMC1326311

[49] Bierhoff H, Schmitz K, Maass F, Ye J, Grummt I (2010) Noncoding transcripts in sense and antisense orientation regulate the epigenetic state of ribosomal RNA genes. Cold Spring Harb Symp Quant Biol 75: 357–364. 21502405 10.1101/sqb.2010.75.060

[50] Servant G, Pinson B, Tchalikian-Cosson A, Coulpier F, Lemoine S, et al (2012) Tye7 regulates yeast Ty1 retrotransposon sense and antisense transcription in response to adenylic nucleotides stress. Nucleic Acids Res 40: 5271–5282. 22379133 10.1093/nar/gks166 PMC3384299

[51] Yassour M, Pfiffner J, Levin JZ, Adiconis X, Gnirke A, et al (2010) Strand-specific RNA sequencing reveals extensive regulated long antisense transcripts that are conserved across yeast species. Genome Biol 11 R87-2010-11-8-r87. Epub 2010 Aug 26. 10.1186/gb-2010-11-8-r87 PMC2945789 20796282

[52] Nakama M, Kawakami K, Kajitani T, Urano T, Murakami Y (2012) DNA-RNA hybrid formation mediates RNAi-directed heterochromatin formation. Genes Cells 17: 218–233. 22280061 10.1111/j.1365-2443.2012.01583.x

[53] Stirling PC, Bloom MS, Solanki-Patil T, Smith S, Sipahimalani P, et al (2011) The complete spectrum of yeast chromosome instability genes identifies candidate CIN cancer genes and functional roles for ASTRA complex components. PLoS Genet 7: e1002057. 21552543 10.1371/journal.pgen.1002057 PMC3084213

[54] Xu Z, Wei W, Gagneur J, Clauder-Munster S, Smolik M, et al (2011) Antisense expression increases gene expression variability and locus interdependency. Mol Syst Biol 7: 468. 21326235 10.1038/msb.2011.1 PMC3063692

[55] Berthelet S, Usher J, Shulist K, Hamza A, Maltez N, et al (2010) Functional genomics analysis of the saccharomyces cerevisiae iron responsive transcription factor Aft1 reveals iron-independent functions. Genetics 185: 1111–1128. 20439772 10.1534/genetics.110.117531 PMC2900968

[56] Li X, Manley JL (2005) Inactivation of the SR protein splicing factor ASF/SF2 results in genomic instability. Cell 122: 365–378. 16096057 10.1016/j.cell.2005.06.008

[57] Strasser K, Masuda S, Mason P, Pfannstiel J, Oppizzi M, et al (2002) TREX is a conserved complex coupling transcription with messenger RNA export. Nature 417: 304–308. 11979277 10.1038/nature746

[58] Huertas P, Aguilera A (2003) Cotranscriptionally formed DNA:RNA hybrids mediate transcription elongation impairment and transcription-associated recombination. Mol Cell 12: 711–721. 14527416 10.1016/j.molcel.2003.08.010

[59] Zenklusen D, Vinciguerra P, Wyss JC, Stutz F (2002) Stable mRNP formation and export require cotranscriptional recruitment of the mRNA export factors Yra1p and Sub2p by Hpr1p. Mol Cell Biol 22: 8241–8253. 12417727 10.1128/MCB.22.23.8241-8253.2002 PMC134069

[60] Rondon AG, Mischo HE, Kawauchi J, Proudfoot NJ (2009) Fail-safe transcriptional termination for protein-coding genes in S. cerevisiae. Mol Cell 36: 88–98. 19818712 10.1016/j.molcel.2009.07.028 PMC2779338

[61] Steinmetz EJ, Warren CL, Kuehner JN, Panbehi B, Ansari AZ, et al (2006) Genome-wide distribution of yeast RNA polymerase II and its control by Sen1 helicase. Mol Cell 24: 735–746. 17157256 10.1016/j.molcel.2006.10.023

[62] Helmrich A, Ballarino M, Tora L (2011) Collisions between replication and transcription complexes cause common fragile site instability at the longest human genes. Mol Cell 44: 966–977. 22195969 10.1016/j.molcel.2011.10.013

[63] Ursic D, Himmel KL, Gurley KA, Webb F, Culbertson MR (1997) The yeast SEN1 gene is required for the processing of diverse RNA classes. Nucleic Acids Res 25: 4778–4785. 9365256 10.1093/nar/25.23.4778 PMC147120

[64] Arana ME, Kerns RT, Wharey L, Gerrish KE, Bushel PR, et al (2012) Transcriptional responses to loss of RNase H2 in saccharomyces cerevisiae. DNA Repair (Amst) 11: 933–941. 23079308 10.1016/j.dnarep.2012.09.006 PMC3535280

[65] Matsuda E, Garfinkel DJ (2009) Posttranslational interference of Ty1 retrotransposition by antisense RNAs. Proc Natl Acad Sci U S A 106: 15657–15662. 19721006 10.1073/pnas.0908305106 PMC2735561

[66] Kawauchi J, Mischo H, Braglia P, Rondon A, Proudfoot NJ (2008) Budding yeast RNA polymerases I and II employ parallel mechanisms of transcriptional termination. Genes Dev 22: 1082–1092. 18413718 10.1101/gad.463408 PMC2335328

[67] Schulze JM, Jackson J, Nakanishi S, Gardner JM, Hentrich T, et al (2009) Linking cell cycle to histone modifications: SBF and H2B monoubiquitination machinery and cell-cycle regulation of H3K79 dimethylation. Mol Cell 35: 626–641. 19682934 10.1016/j.molcel.2009.07.017 PMC3222332

[68] van Bakel H, van Werven FJ, Radonjic M, Brok MO, van Leenen D, et al (2008) Improved genome-wide localization by ChIP-chip using double-round T7 RNA polymerase-based amplification. Nucleic Acids Res 36: e21. 18180247 10.1093/nar/gkm1144 PMC2275083

[69] Holstege FC, Jennings EG, Wyrick JJ, Lee TI, Hengartner CJ, et al (1998) Dissecting the regulatory circuitry of a eukaryotic genome. Cell 95: 717–728. 9845373 10.1016/s0092-8674(00)81641-4

[70] Kinsella RJ, Kahari A, Haider S, Zamora J, Proctor G, et al (2011) Ensembl BioMarts: A hub for data retrieval across taxonomic space. Database (Oxford) 2011: bar030. 21785142 10.1093/database/bar030 PMC3170168

[71] Hentrich T, Schulze JM, Emberly E, Kobor MS (2012) CHROMATRA: A galaxy tool for visualizing genome-wide chromatin signatures. Bioinformatics 28: 717–718. 22238257 10.1093/bioinformatics/bts007

[72] Michaelis C, Ciosk R, Nasmyth K (1997) Cohesins: Chromosomal proteins that prevent premature separation of sister chromatids. Cell 91: 35–45. 9335333 10.1016/s0092-8674(01)80007-6

[73] Klein F, Laroche T, Cardenas ME, Hofmann JF, Schweizer D, et al (1992) Localization of RAP1 and topoisomerase II in nuclei and meiotic chromosomes of yeast. J Cell Biol 117: 935–948. 1315786 10.1083/jcb.117.5.935 PMC2289479

[74] Plenary Sessions. Yeast 30: 21–43 doi10.1002yea.2971/yea.2971

[75] Gavalda S, Gallardo M, Luna R, Aguilera A (2013) R-loop mediated transcription-associated recombination in trf4Delta mutants reveals new links between RNA surveillance and genome integrity. PLoS One 8: e65541. 23762389 10.1371/journal.pone.0065541 PMC3676323

[76] Gallardo M, Luna R, Erdjument-Bromage H, Tempst P, Aguilera A (2003) Nab2p and the Thp1p-Sac3p complex functionally interact at the interface between transcription and mRNA metabolism. J Biol Chem 278: 24225–24232. 12702719 10.1074/jbc.M302900200

[77] Gonzalez-Aguilera C, Tous C, Gomez-Gonzalez B, Huertas P, Luna R, et al (2008) The THP1-SAC3-SUS1-CDC31 complex works in transcription elongation-mRNA export preventing RNA-mediated genome instability. Mol Biol Cell 19: 4310–4318. 18667528 10.1091/mbc.E08-04-0355 PMC2555943

[78] Santos-Pereira JM, Herrero AB, Garcia-Rubio ML, Marin A, Moreno S, et al (2013) The Npl3 hnRNP prevents R-loop-mediated transcription-replication conflicts and genome instability. Genes Dev 27: 2445–2458. 24240235 10.1101/gad.229880.113 PMC3841734

